# Limited fusion strategy for congenital scoliosis: is it truly one and done?

**DOI:** 10.1007/s43390-026-01326-w

**Published:** 2026-03-10

**Authors:** Brandon Yoshida, Tyler A. Tetreault, Luke Drake, Tiffany Phan, Jacquelyn Valenzuela-Moss, Tishya A. L. Wren, Lindsay M. Andras, Michael J. Heffernan

**Affiliations:** 1 Keck School of Medicine, University of Southern California, Los Angeles, CA USA; 2 Jackie and Gene Autry Orthopedic Center, Children’s Hospital Los Angeles, 4650 Sunset Blvd, Mailstop #69, Los Angeles, CA 90027 USA; 3 Department of Orthopaedic Surgery, University of Mississippi Medical Center, Jackson, MS USA; 4 Alfred E. Mann Department of Biomedical Engineering, Viterbi School of Engineering, University of Southern California, Los Angeles, CA USA

**Keywords:** Congenital scoliosis, Fusion, Reoperation, End vertebra

## Abstract

**Purpose:**

To investigate the rate and risk factors for reoperation following a short-segment fusion strategy for congenital scoliosis (CS).

**Methods:**

CS patients treated with posterior fusion were identified and stratified by history of revision surgery. Demographic and pre- and postoperative radiographic data were compared to determine the risk factors for reoperation.

**Results:**

Thirty-five patients (mean age 5.0 years; mean follow-up 7.6 years) underwent an average of 3.5 levels fused. Index procedures were performed in the thoracic (49%), thoracolumbar (29%), lumbar (17%), and lumbosacral (6%) regions. Eighteen (51%) patients underwent reoperation at a mean of 5.2 years. Preoperative curve magnitude was significantly lower in patients who did not require reoperation (34.2° vs 66.7°, *p* < 0.001), with preoperative curves ≥ 45° having a 94% reoperation rate compared to 6% in curves < 45°. Preoperative curve magnitude strongly predicted reoperation (AUC 0.99, 95% CI 0.97–1.00), with 45° as the optimal cutoff (94.4% sensitivity, 94.1% specificity). Larger pre- and postoperative compensatory curves and constructs that did not span both end vertebrae were also risk factors for reoperation (*p* < 0.05). In multivariate analysis, only greater preoperative curve magnitude retained significance for reoperation (*p* = 0.028).

**Conclusions:**

Although short-segment fusion for CS resulted in a 51% reoperation rate, stabilization for 6.2 years with this staged strategy allowed for growth and delayed additional surgery to a later, optimal age. Risk factors for reoperation included preoperative curves ≥ 45° and constructs failing to span both end vertebrae.

**Level of Evidence:**

III.

## Introduction

Congenital scoliosis is a spinal deformity caused by improperly formed vertebrae. This occurs early in development and is present at birth, although subsequent diagnosis may not occur until much later. Additionally, this patient population is heterogeneous, with variations including but not limited to the degree of deformity, location, type of anomaly, and age at presentation. [1, 2] As such, the treatment of congenital scoliosis and the timing of surgery are also heterogeneous without clear consensus.

In general, approximately 25% of congenital scoliosis cases do not progress, while 25% progress mildly, and 50% require treatment. [3] Among those that do require treatment, the timing of surgery is variable due to the competing interests of curve progression prevention in earlier surgery versus the downsides of early fusion in the growing spine and the potential for reoperation. [4, 5] In addition to the heterogeneity regarding the timing of surgery, there remains significant controversy among the available surgical treatment options including but not limited to in situ fusion, hemiepiphysiodesis, hemivertebrectomy, and growing rods. [6]

Short-segment (≤ 6 segments) fusion constructs are a common approach in the management of congenital scoliosis. [7] However, short-segment constructs may require reoperation in the form of growing rods or longer fusions secondary to adding-on or progression of compensatory curves. [8] Little is known with regard to optimal patient selection for short-segment constructs nor the risk factors associated with revision surgery. The purpose of this study was to investigate the rate and risk factors for reoperation following a short-segment fusion strategy for congenital scoliosis. We hypothesized that patient and curve characteristics would impact the rate of reoperation in this cohort.

## Methods

### Study design and participants

Following institutional review board approval, a single-center retrospective chart review was performed of patients who underwent short fusion between January 2004 and May 2022. CPT codes for short fusion and excision of vertebral body were queried including the following: 22,800, 22,808, 22,110, 22,112, and 22,114. Short fusion was defined as a fusion of 6 levels or less.

**Fig. 1:**
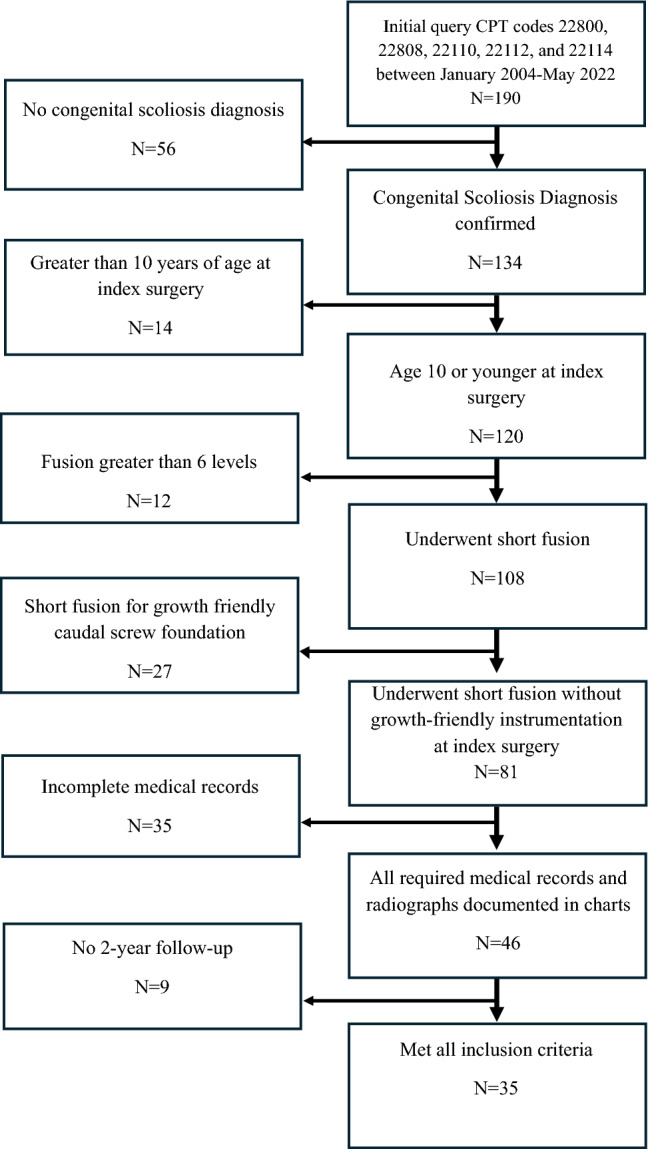
Flowchart of patient selection

Patients were included if they were 10 years of age or younger at the time of the index procedure, had a confirmed diagnosis of congenital scoliosis, and had radiographs available at the following timepoints: preoperative, postoperative, and ≥ 2-year follow-up. Patients were excluded if they were over the age of 10 at the time of the index procedure, had a scoliosis diagnosis other than congenital, had a history of previous spine surgery, or underwent other surgical strategies such as growth-friendly instrumentation. Patients were also excluded if radiographs were not available before and after the index procedure and at a minimum of 2 years following the index surgery. (Fig. 1) Reoperation was defined as any return to the operating room, including planned and unplanned surgeries.

### Data collection

Demographic data including age, sex, race, height, weight, and medical comorbidities were collected as baseline information for each patient. Treatment information collected included the following: preoperative bracing/casting, index surgical procedure details, complications, and the details of any additional surgical procedures during follow-up. Radiographic measurements included pre- and postoperative coronal curve magnitude, pre- and postoperative kyphosis and lordosis, coronal and sagittal balance, total spine length, number, type, and location of congenital anomalies, location of the end vertebrae, compensatory curve magnitude, as well as tilt of the upper and lower instrumented levels. Radiographic evaluation was performed by two physician members of the research team not associated with the clinical evaluation process, who reviewed sequential radiographs for all patients in a blinded fashion at the time of the study. The surgeries were performed by one of four fellowship-trained pediatric spine surgeons.

### Data analysis

Data were analyzed using STATA version 14.2 (StataCorp LP, College Station, TX) with a significance level of 0.05. Descriptive data were reported using means and standard deviations for continuous variables and counts and frequencies for categorical data. Fisher’s exact test and 2-sided independent *t* tests were used to compare demographic, radiographic, and surgical variables between patients who required additional surgery to those who did not.

Reviewer operating characteristic (ROC) analysis was performed to identify the optimal preoperative curve magnitude threshold for predicting reoperation. Sensitivity and specificity were calculated across all possible values of preoperative curve magnitudes, and the cutoff was determined utilizing the value with the greatest Youden Index. Predictive performance was evaluated using the area under the curve (AUC) with asymptotic standard error and 95% confidence intervals.

Univariate associations were assessed using standard logistic regression models to determine the odds ratios for the need for additional surgeries by curve severity. Due to the small sample size and quasi-complete separation observed in preliminary multivariate models, a Firth-penalized logistic regression was utilized to estimate the adjusted odds ratios in the multivariate model. This approach applies a penalized maximum likelihood correction that reduces small-sample biases [9].

Lastly, a sensitivity analysis was performed to assess the robustness of the findings and to evaluate whether patients with non-consecutive short fusions disproportionately influenced the results. Patients with two non-consecutive short fusions (*N* = 4) were excluded from the primary analytic cohort, and all models were re-run. Effect estimates were compared with the primary analysis to assess for meaningful changes in magnitude or direction of the associations. A change ≥ 15% in effect estimates was considered a meaningful deviation from the primary findings.

## Results

**Fig. 2:**
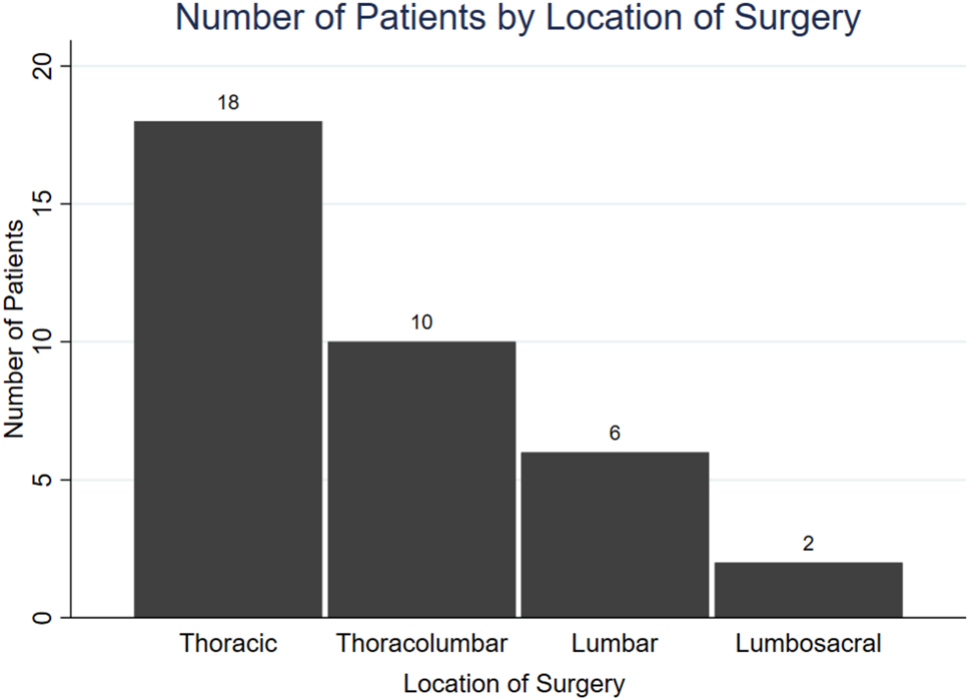
Number of patients by location of surgery

A total of 35patients were included for analysis with a mean ± standard deviation (SD) age of 5.0 ± 2.5 years. Average follow-up was 7.6 ± 3.4 years. All 35 patients underwent short fusion (≤ 6 levels), of which 60% (21/35) included hemivertebrectomy and 40% (14/35) included osteotomies (Table 1). Forty-six percent of patients underwent surgery in the thoracic region, 31% in the thoracolumbar, 17% in the lumbar, and 6% in the lumbosacral region (Fig. 2). The mean number of fused levels was 3.5 ± 1.4 levels. Four (11.4%) patients had two areas of fusion (separated by unfused segments) at the time of the index procedure.

**Table 1:** Index procedure details

Procedure	All participants *N* = 35	Did not need additional surgery, *N* = 17	Needed additional surgery, *N* = 18	*P* value
Hemivertebrectomy	21 (60%)	8 (47.1%)	13 (72.2%)	0.176
Short fusion	35 (100%)	17 (100%)	18 (100%)	–
Number of levels fused	3.5 (1.4)	3.3 (1.2)	3.7 (1.5)	0.43
Osteotomy	14 (40%)	8 (47.1%)	6 (33.3%)	0.500
Instrumentation				
Non-instrumented	0	–	–	–
Wires	1 (2.9%)	0	1 (5.6%)	> 0.999
Hooks	18 (51.4%)	7 (41.2%)	11 (61.1%)	0.318
Pedicle screws	35 (100%)	17 (100%)	18 (100%)	–
All congenital anomalies addressed during index procedure	28 (80.0%)	13 (76.5%)	15 (83.3%)	0.69

**Fig. 3:**
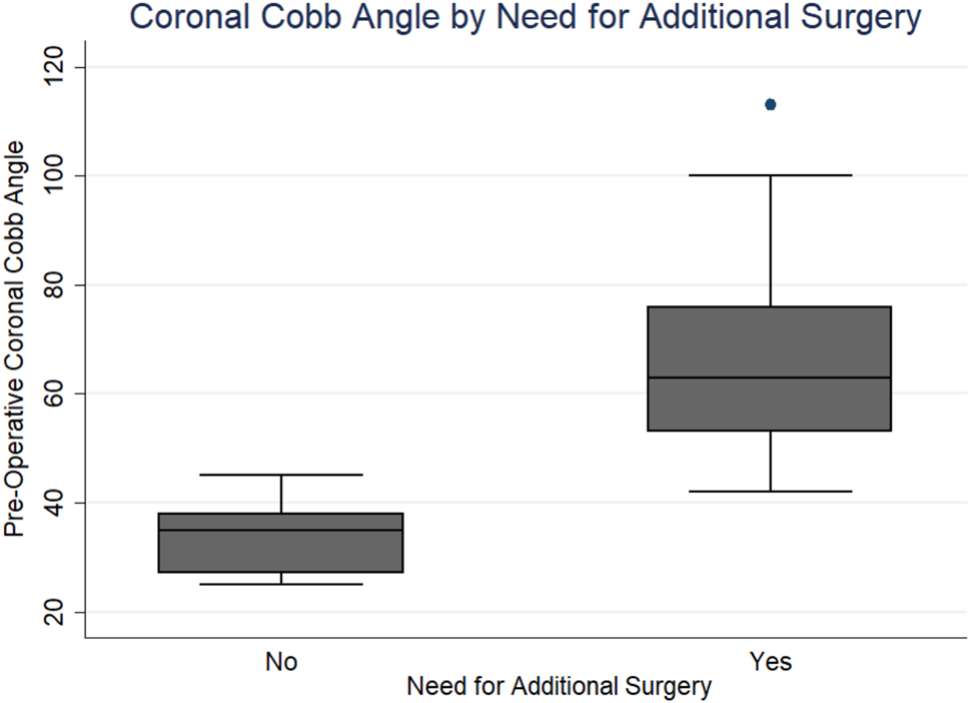
Coronal Cobb angle by need for additional surgery

Eighteen (51.4%) patients underwent a mean number of 1.8 ± 3.0 additional surgeries. Ten of the eighteen patients (55.6%) underwent a revision of their index PSF, 6/18 (33.3%) underwent irrigation and debridement, and another 6/18 (33.3%) required implant exchange. In addition, 4/18 (22.2%) ultimately required conversion to growth-friendly instrumentation. All additional surgeries are listed in Table 2. Among patients requiring additional surgery, the mean interval from short fusion to subsequent curve correction treatment (i.e., fusion revision/additional fusion, growth-friendly implantation, bracing) was 6.2 ± 3.4 years. Compared to the cohort of 17 (48.5%) patients who did not undergo additional surgery, preoperative curve magnitude was significantly greater (66.7° ± 20.3° vs. 34.0° ± 6.7°; *p* < 0.001) in the group undergoing additional surgery. Postoperative curve magnitude was 37.7° ± 26.7° in the group who required additional surgery compared to 20.9° ± 13.7° in patients who did not require additional surgery (*p* = 0.03) (Fig. 3). Reoperation was associated with greater compensatory curve magnitude both preoperatively (35.9 ± 12.9 vs 20.5 ± 11.8, *p* = 0.006) and postoperatively (24.9 ± 14.0 vs 9.1 ± 6.7, *p* = 0.007). Moreover, failure of the upper instrumented vertebra (UIV) and/or lowest instrumented vertebra (LIV) to span the end vertebra (EV) was significantly associated with need for additional surgery (*p* = 0.04). All other demographic and operative variables were not significantly different between the two groups (*p* > 0.05) (Table 3).

**Table 2:** Patient-level breakdown of additional surgeries

Procedure type	Count (*N* = 18)^a^
Revision of index PSF	10 (55.6%)
Implant exchange	6 (33.3%)
Irrigation and debridement (I&D)	6 (33.3%)
Conversion to PSF after removal of growth-friendly instrumentation	4 (22.2%)
Broken growth-friendly instrumentation	2 (11.1%)
Growth-friendly lengthening	4 (22.2%)
Casting	1 (5.6%)
Other	7 (38.9%)

^a^Results are reported as *N* (%). Nine patients had more than one surgery

**Table 3:** Patient demographics and clinical characteristics

	All participants (*N* = 35)	Did not need additional surgery (*N* = 17)	Needed additional surgery (*N* = 18)	*P* value
Age (yr)	5.0 (2.5)	5.4 (3.2)	4.6 (1.5)	0.32
Sex				0.31
Female	14 (40.0%)	5 (29.4%)	9 (50.0%)	
Male	21 (60.0%)	12 (70.6%)	9 (50.0%)	
Race/Ethnicity				0.12
Latinx	9 (25.7%)	5 (29.4%)	4 (22.2%)	
Non-Hispanic White	18 (51.4%)	10 (58.8%)	8 (44.4%)	
Other	5 (14.3%)	0	5 (27.8%)	
Not specified	3 (8.6%)	2 (11.8%)	1 (5.6%)	
Fused ribs	5 (16.1%)	2 (13.3%)	3 (18.8%)	0.53
Syndromic etiology	19 (54.3%)	9 (52.9%)	10 (55.6%)	1.00
Length of follow-up (yr)	7.6 (3.4)	6.0 (2.8)	9.1 (3.4)	0.006
Age at final follow-up (yr)	12.6 (3.6)	11.4 (3.3)	13.7 (3.5)	0.06
Comorbidities	22 (62.9%)	11 (64.7%)	11 (61.1%)	1.00
Number of hemivertebra				
Single	17 (48.6%)	9 (52.9%)	8 (44.4%)	0.74
Multiple	18 (51.4%)	8 (47.1%)	10 (55.6%)	
Hemivertebra segmentation				
Fully segmented	24 (68.6%)	12 (70.6%)	12 (66.7%)	1.00
Partially segmented	7 (20.0%)	3 (17.7%)	4 (22.2%)	1.00
Unsegmented	4 (11.4%)	2 (11.8%)	2 (11.1%)	1.00
Locations affected				
Thoracic	17 (48.6%)	8 (47.1%)	9 (50.0%)	0.58
Thoracolumbar (T11-L1)	10 (28.6%)	5 (29.4%)	5 (27.8%)	
Lumbar	6 (17.1%)	2 (11.7%)	4 (22.2%)	
Lumbosacral (L5-Sacrum)	2 (5.7%)	2 (11.7%)	–	
Pre-op curve magnitude (deg)	**50.9 (22.3)**	**34.2 (6.7)**	**66.7 (20.3)**	** < 0.0001**
Percent curve correction (%)	43.8 (33.8)	40.0 (37.5)	47.4 (30.7)	0.52
Post-op curve magnitude (deg)	**29.6 (22.8)**	**20.9 (13.7)**	**37.7 (26.7)**	**0.03**
Pre-op compensatory curve (deg)	**28.7 (14.5)**	**20.5 (11.8)**	**35.9 (12.9)**	**0.001**
Post-op compensatory curve (deg)	**16.9 (13.3)**	**9.1 (6.7)**	**24.3 (13.9)**	**0.0002**
EV Level	11 (31.4%)	8 (47.1%)	3 (16.7%)	0.08
UIV and LIV Span EV	**14 (40.0%)**	**10 (58.8%)**	**4 (22.2%)**	**0.04**

Continuous variables are presented as mean (SD), categorical variables as *n* (%)

Statistically significant results are bolded

Importantly, preoperative curves ≥ 45° had a 94% (17/18) reoperation rate compared to curves < 45° with a reoperation rate of 6% (1/17) (*p* < 0.001). The ROC analysis demonstrated that preoperative curve magnitude strongly predicted risk of reoperation (AUC 0.99, 95% CI [0.97–1.00]). The optimal cutoff identified using the Youden Index was 45°, with a sensitivity of 94.4% and specificity of 94.1% in identifying patients who ultimately required reoperation. In the univariate logistic regression model, this association exhibited evidence of quasi-complete separation, resulting in inflated odds ratios (OR 272, 95% CI [15.7–4724.2], *p* < 0.001), that could not be interpreted reliably. However, spanning the EV with the UIV and/or LIV was associated with an approximate 80% decrease in odds of requiring additional surgery (OR 0.20; 95% CI [0.05–0.87], *p* = 0.032). Additionally, greater compensatory curve magnitude was associated with higher odds of additional surgery, increasing by 11% per degree preoperatively (OR 1.11, 95% CI [1.03–1.19], *p* = 0.006) and 19% per degree postoperatively (OR 1.19; 95% CI [1.05–1.35], *p* = 0.007). Variables with a *p* value < 0.10 in the univariate logistic regression were included in the multivariate model. In the multivariate Firth logistic regression analysis accounting for significant predictors from the univariate analysis, only preoperative curve magnitude remained a significant predictor of the need for additional surgery (OR 1.27, 95% CI [1.01–1.61], *p* = 0.045) (Table 4). The overall model fit was not statistically significant (*p* = 0.40), consistent with a limited sample size.

**Table 4:** Univariate logistic regression model for odds of reoperation

Variable	Unadjusted odds ratio	95% confidence interval	*P* value
Univariate logistic regression models with unadjusted odds ratios			
Preoperative curve magnitude	1.84	0.90–3.74	0.094
Preoperative compensatory curve	1.11	1.03–1.19	0.006
Postoperative curve magnitude	1.04	1.00–1.08	0.041
Postoperative compensatory curve	1.19	1.05–1.35	0.007
Percent curve correction	1.01	0.99–1.03	0.512
Spanning UIV/LIV	0.20	0.05–0.87	0.032
EV Level	0.23	0.05–1.07	0.061
Multivariate Firth logistic regression with adjusted odds ratios			
Preoperative curve magnitude	1.28	1.03–1.59	0.028
Preoperative compensatory curve	0.95	0.81–1.10	0.481
Postoperative curve magnitude	0.97	0.88–1.06	0.493
Postoperative compensatory curve	1.12	0.91–1.40	0.288
Spanning UIV/LIV	0.60	0.04–8.83	0.712
EV level	3.17	0.06–173.79	0.572

Overall model Wald *χ*^2^ = 6.20, *p* = 0.4011, penalized log likelihood = 5.9329

Results of the sensitivity analysis were consistent with the primary findings. Exclusion of patients with non-consecutive short fusion did not meaningfully alter the direction or magnitude of effect estimates; all previously significant variables remained significant, as changes in estimates did not surpass a 15% threshold.

## Discussion

This study sought to investigate the rate and the risk factors for reoperation after short-segment posterior fusion constructs (≤ 6 levels) for congenital scoliosis. Preoperative and postoperative curves’ magnitude as well as the status of compensatory curves were important risk factors for reoperation after implementation of a short-segment fusion strategy. ROC analysis showed that preoperative curve magnitude was a strong predictor of reoperation risk. The Youden Index identified 45° as the optimal cutoff, with 94.4% sensitivity and 94.1% specificity. Notably, patients with preoperative curves ≥ 45° had a 94% reoperation rate, compared with only 6% for those with curves < 45°. Our results also suggest that inclusion of the EV is important to mitigate additional surgery. The findings of this study have implications for decision-making in terms of surgical timing as well as surgical strategy.

Although there has been equipoise regarding the optimal timing of surgery, some recent literature has promoted earlier operative management of congenital scoliosis. [7, 8, 10] The unifying theme of these studies is patient age at the time of the index procedure. A recent single-center retrospective study by Lin et al. reviewed ten-year trends in 1,207 congenital scoliosis cases undergoing operative management and noted an increasing trend toward earlier operative intervention, particularly in patients aged between 0 and 5 years. [7] Recent studies have also demonstrated improved deformity correction without increased complication rates when corrective surgery for congenital scoliosis is performed at an earlier age. [1, 3, 11] Chang et al. [1] reviewed 18 patients with congenital scoliosis who underwent surgery under the age of 10 and found that surgery before the age of 6 had significantly greater deformity correction at final follow-up without any significant difference in crankshaft phenomena or other complications. However, early surgical intervention for congenital scoliosis is not without risk. Ruf and Harms [12] reported a 28.5% complication rate in a cohort with a mean age of 3 years undergoing posterior hemivertebrectomy with fusion. Reported complications included two pedicle fractures, three instrumentation failures, two reoperations for curve progression, and one postoperative infection. More recently, Oksanen et al. [13] demonstrated a 27% complication rate associated with early hemivertebrectomy and short-segment fusion constructs, including a 10% incidence of deep surgical site infection and a 10% rate of reoperation. In a meta-analysis, Barik et al. found only younger age was a significant independent risk factor for revision surgery, with a mean age of 5.9 ± 4 years in the cohort requiring revision surgery compared to 8.4 ± 4.7 years in the group not requiring revision surgery. [14] Therefore, there is currently equipoise with regard to recommendations for age-based surgical timing. Interestingly, a closer look reveals that the average preoperative curve magnitude in successful patients was relatively small in these studies. The data in our study are consistent with previous work; the difference is the recognition that preoperative curve magnitude and not age is likely the best predictor of success. Certainly, there is an overlap between age and curve magnitude, but recommendations based on age alone may lead to inconsistent results. The data from our study suggest that short-segment posterior fusion constructs (≤ 6 levels) should occur before the curve reaches 45 degrees. Surgeons should have a specific focus on curve magnitude when following patients with congenital scoliosis if planning a short-segment strategy.

In our study, the primary risk factor for reoperation after short-segment posterior fusion was preoperative curve magnitude. In other studies, age, bisegmental fusions [15] and thoracic location [16] also appeared to be risk factors for revision surgery. Although the patients in our cohort that underwent reoperation were slightly younger and had a slightly higher relative percentage of thoracic hemivertebrae, none of the existing risk factors for revision surgery were found to be significant within our study. Further discrepancies can be found when looking at overall reoperation rates, which was 7.8% in the meta-analysis by Barik et al. compared to 52.8% in our study. Although numerous reasons can explain this discrepancy, such as publication bias, one primary explanation is the difference in preoperative curves within existing literature compared to our study. Across the literature, the majority of preoperative scoliosis curves measure approximately 30°–40°. [1, 11, 14, 17] Although this is consistent with the curves in our cohort that did not require reoperation, the curve magnitude in patients requiring reoperation is almost twice that found in the literature. This emphasizes the importance of intervening at smaller preoperative curve magnitudes to minimize the need for reoperation.

Our study also found failure to span the EV as another risk factor for reoperation after a short-segment fusion for congenital scoliosis. In our findings, 58.8% of patients who did not require additional surgery had a construct that spanned the cephalad and caudal EV compared to 22.2% in patients who underwent revision surgery. To our knowledge, this is an under-investigated finding, as the current literature has limited evidence on construct length as a risk factor for revision surgery after posterior fusion for congenital scoliosis. The only relevant evidence for patients with congenital scoliosis is from the meta-analysis by Barik et al. [14] who reports a mean number of fusion levels of 3.5 ± 1.4 associated with a significant correlation between number of levels fused and increased correction rate, blood loss, and operative time after hemivertebrectomy and posterior fusion. More broadly, another meta-analysis by Yang et al. [18] on idiopathic scoliosis found that failure to span the caudal EV significantly increased the risk of the “adding-on” phenomenon, often necessitating revision surgery and caudal extension of the fusion construct. Although further work is still needed, our findings are a necessary first step to optimize short-segment posterior fusion (≤ 6 levels) construct indications for patients with congenital scoliosis and weigh the benefits of increased correction rate and decreased revision surgery rates with worse blood loss, operative time, and implant cost.

An important finding of our study was the association between postoperative compensatory curves and the need for revision surgery following hemivertebrectomy and short-segment fusion on univariate analysis. This suggests that adequate correction of the primary curve alone may not suffice if compensatory curves are not appropriately addressed, potentially leading to the development of emerging scoliosis (ES) defined as an apical vertebra located ≥ 2 levels from the fused area with an increase in the Cobb angle of at least 20° from baseline. [19] Li et al. [20] reported that 20% of patients with congenital cervicothoracic scoliosis who underwent posterior hemivertebrectomy and fusion-developed ES, with some necessitating further surgical intervention. While compensatory curves generally improve by approximately 30–70% following short fusion constructs of the primary curve, [21, 22] postoperative compensatory curve magnitude remains an independent risk factor for ES in the literature. [19] Consistent with our findings, patients exhibiting higher postoperative compensatory curves are at increased risk for revision surgery, although this was demonstrated only on univariate analysis. While further studies may be necessary to further elucidate the effect of residual compensatory curve on reoperation rates, our study suggests that compensatory curve may be a proxy for larger or more ominous curves that require more mindful preoperative planning. Thus, surgeons should carefully consider the importance of predicting response of the compensatory curves to the short-segment fusion plan. A short-segment strategy may not be the optimal approach in some patients with substantial compensatory curves above or below the planned construct levels.

Our study is not without limitations. The retrospective nature of our study may introduce selection bias and confounding variables that are not accounted for in our analysis. Second, our sample size is relatively small and heterogeneous consisting of only 35 patients of varying preoperative curve magnitudes and medical comorbidities. Our study does not include patient-reported outcomes. Also, the evaluation of reoperation rates may be influenced by the follow-up duration, although we had similar follow-up times in both groups. This study does have numerous strengths including the length of follow-up, which surpasses the 5-year follow-up period typical of other studies. Additionally, our preoperative curve magnitude is greater than that of existing studies, which is pivotal in revealing a risk factor for reoperation that has not been previously discussed.

Short-segment posterior fusion constructs (≤ 6 levels) for congenital scoliosis are an effective surgical treatment for congenital scoliosis, but these constructs are not necessarily definitive treatment for some patients. On univariate analysis, preoperative curve magnitude, relative curve correction, and compensatory curve severity were risk factors for reoperation, but only preoperative curve magnitude retained significance in multivariate analysis.

Specifically, patients with a preoperative curve magnitude ≥ 45° had a significantly higher rate of revision surgery, with this cutoff demonstrating both high sensitivity and specificity for predicting reoperation risk. Although indications and timing for operative management of congenital scoliosis are multifactorial, short-segment fusion constructs (≤ 6 levels) as performed in this cohort should be considered for smaller curve magnitudes while alternative approaches such as growth-friendly implants should be considered for curves of greater magnitude.

## Data Availability

Data utilized for this study is available upon request.
